# Serum lipoproteins attenuate macrophage activation and Toll-Like Receptor stimulation by bacterial lipoproteins

**DOI:** 10.1186/1471-2172-11-46

**Published:** 2010-09-16

**Authors:** Sylvette Bas, Richard W James, Cem Gabay

**Affiliations:** 1 Division of Rheumatology, Department of Internal Medicine, Geneva University Hospital, 1211 Geneva 14, Switzerland; 2 Division of Rheumatology, Department of Genetics and Laboratory Medicine, Geneva University Hospital, 1211 Geneva 14, Switzerland; 3 Lipoprotein Research Group, Clinical Diabetes Unit, Department of Internal Medicine, Geneva University Hospital, Switzerland; 4 Lipoprotein Research Group, Clinical Diabetes Unit, Department of Genetics and Laboratory Medicine, Geneva University Hospital, Switzerland; 5 Division of Rheumatology, Department of Pathology and Immunology, Geneva University Hospital, Switzerland

## Abstract

**Background:**

*Chlamydia trachomatis *was previously shown to express a lipoprotein, the macrophage infectivity potentiator (Mip), exposed at the bacterial surface, and able to stimulate human primary monocytes/macrophages through Toll Like Receptor (TLR)2/TLR1/TLR6, and CD14. In PMA-differentiated THP-1 cells the proinflammatory activity of Mip was significantly higher in the absence than in the presence of serum. The present study aims to investigate the ability of different serum factors to attenuate Mip proinflammatory activity in PMA-differentiated THP-1 cells and in primary human differentiated macrophages. The study was also extend to another lipoprotein, the *Borrelia burgdorferi *outer surface protein (Osp)A. The proinflammatory activity was studied through Tumor Necrosis Factor alpha (TNF-α) and Interleukin (IL)-8 release. Finally, TLR1/2 human embryonic kidney-293 (HEK-293) transfected cells were used to test the ability of the serum factors to inhibit Mip and OspA proinflammatory activity.

**Results:**

In the absence of any serum and in the presence of 10% delipidated FBS, production of Mip-induced TNF-α and IL-8 in PMA-differentiated THP-1 cells were similar whereas they were significantly decreased in the presence of 10% FBS suggesting an inhibiting role of lipids present in FBS. In the presence of 10% human serum, the concentrations of TNF-α and IL-8 were 2 to 5 times lower than in the presence of 10% FBS suggesting the presence of more potent inhibitor(s) in human serum than in FBS. Similar results were obtained in primary human differentiated macrophages. Different lipid components of human serum were then tested (total lipoproteins, HDL, LDL, VLDL, triglyceride emulsion, apolipoprotein (apo)A-I, B, E2, and E3). The most efficient inhibitors were LDL, VLDL, and apoB that reduced the mean concentration of TNF-α release in Mip-induced macrophages to 24, 20, and 2%, respectively (*p *< 0.0001). These lipid components were also able to prevent TLR1/2 induced activation by Mip, in HEK-293 transfected cells. Similar results were obtained with OspA.

**Conclusions:**

These results demonstrated the ability of serum lipids to attenuate proinflammatory activity of bacterial lipoproteins and suggested that serum lipoproteins interact with acyl chains of the lipid part of bacterial lipoproteins to render it biologically inactive.

## Background

Among the bacterial components that trigger macrophage activation, the most widely studied is lipopolysaccharide (LPS) but bacterial lipoproteins have also been implicated in inflammatory processes [1-3]. Bacterial lipoproteins are characterized by a unique amino-terminal lipo-amino acid, *N*-acyl-*S*-diacylglyceryl cysteine [4], and this lipid element and its peptide moieties are known to be critical for cell activation through TLR2 [5]. In *C. trachomatis*, one such lipoprotein, the macrophage infectivity potentiator (Mip), has been shown to be present at the bacterial surface [6] and to stimulate the proinflammatory cytokine response to *C. trachomatis *in human macrophages through toll like receptor (TLR)2/TLR1/TLR6 and CD14. The lipid part of Mip has also been shown to be responsible for its proinflammatory activity [2]. However, when stimulation of PMA-differentiated THP-1 cells was performed in the presence of serum, the Mip-induced TNF-α production was significantly decreased [2]. Whereas physiological levels of serum lipoproteins: HDL, LDL, and VLDL have been found to inactivate LPS [7,8] and bacterial lipoteichoic acid [9], no study has been reported so far about the potential of serum lipoproteins to neutralize bacterial lipoproteins. Their ability to neutralize Mip proinflammatory activity was therefore investigated. The study was also extended to another lipoprotein, the *Borrelia burgdorferi *outer surface protein (Osp)A. The results of the studies included herein showed that total lipoproteins, HDL, LDL, VLDL, as well as different apolipoproteins and triglycerides prevented proinflammatory activity of Mip and OspA through TLR1/2.

## Results

### Human serum prevented proinflammatory activity of Mip in PMA-differentiated THP-1 cells

**Figure 1:**
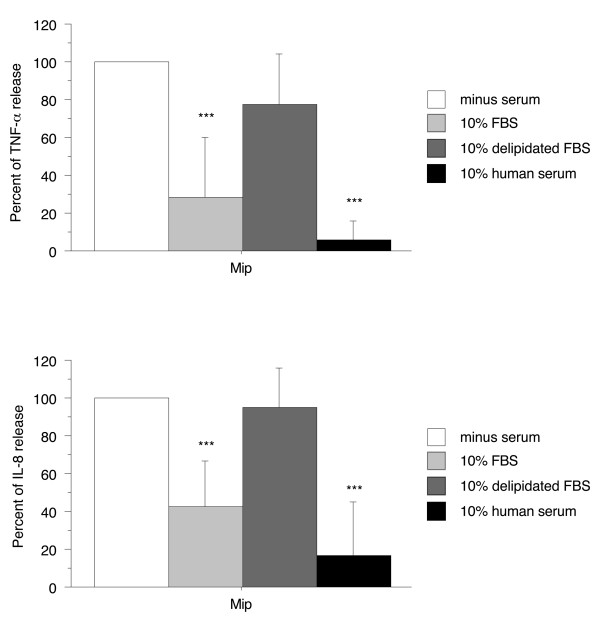
**Effect of 10% FBS, delipidated FBS, and human serum on the ability of Mip to induce TNF-α and IL-8 production by PMA-differentiated THP-1 cells**. Cells (2.5 × 10^5 ^cells/1 ml per well) were cultured with 10 nM PMA for 48 h and then stimulated by recombinant Mip (1 μg/ml) in the presence or absence of 10% heat-inactivated serum. After 4 h stimulation, supernatants were collected and their content in TNF-α and IL-8 were analyzed by ELISA. Each value represents the mean ± SD of triplicates from four experiments. ***: *p *< 0.0001 determined by comparison with medium alone using Student's t test.

In a previous report, we showed that TNF-α production induced by recombinant Mip lipoprotein in PMA-differentiated THP-1 cells was significantly lower in the presence than in the absence of 10% FBS [2]. To extend this observation to another inflammatory cytokine (IL-8) and to test the effect of other sera, Mip-induced TNF-α and IL-8 productions were examined in the presence of 10% delipidated FBS or human serum. To normalize different experiments for day to day variations in the amounts of cytokine produced, data are reported as percent of the cytokine concentration found in the absence of serum with 100% corresponding to a median TNF-α concentration of 0.97 ng/ml (interquartile range (IQR) 0.66 - 1.7) and to a median IL-8 concentration of 1.5 ng/ml (IQR 0.56 - 3.2). Whereas in the presence of 10% FBS the concentrations of TNF-α and IL-8 were significantly lower than in the absence of serum (28 ± 32% and 42 ± 24%, respectively, *p *< 0.0001), no significant difference was observed in the presence of delipidated FBS, suggesting an inhibiting role of lipids present in FBS. In the presence of 10% human serum, the concentrations of TNF-α and IL-8 were also significantly lower than in the absence of serum (6 ± 9% and 17 ± 28%, respectively, *p *< 0.0001) and the mean percentages were 2 to 5 times lower than in the presence of 10% FBS suggesting the presence of more potent inhibitor(s) in the human serum than in the FBS (Figure 1). The fact that the LDL level is 5-6 times lower in FBS than in human serum [10] and that FBS does not contain VLDL [10,11] in contrast to human serum might explain these results.

### Human serum prevented proinflammatory activity of Mip in primary human differentiated macrophages

**Figure 2:**
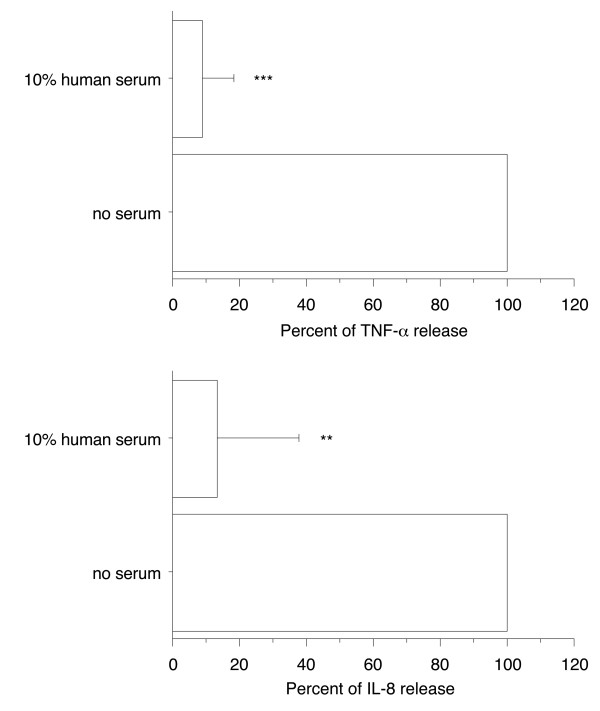
**Effect of 10% human serum on the ability of Mip to induce TNF-α and IL-8 production by primary human differentiated macrophages**. Human monocytes/macrophages (5 × 10^5 ^cells/0.5 ml per well) were differentiated in a 6-day culture in the presence of 2 ng/ml M-CSF and then stimulated by recombinant Mip (1 μg/ml) in the presence or absence of 10% heat-inactivated human serum. After 4 h stimulation, supernatants were collected and their content in TNF-α and IL-8 were analyzed by ELISA. Each value represents the mean ± SD of triplicates from two experiments. The results are expressed as percent of TNF-α or IL-8 release in the absence of serum. ***: *p *< 0.0001; **: *p *< 0.005 determined by comparison with medium alone using Student's t test.

To extend our observations to more physiological cells, the effect of 10% human serum was examined in primary human differentiated macrophages. The concentrations of TNF-α and IL-8 were determined in the cell supernatants after 4 h activation with 1 μg/ml of recombinant Mip in the presence or absence of 10% human serum. To normalize different experiments for day to day variations in the amounts of cytokines produced, data are reported as percent with 100% corresponding to a median TNF-α concentration of 0.4 ng/ml (IQR 0.2 - 0.9) and a median IL-8 concentration of 5.8 ng/ml (IQR 5.0 - 7.0). The presence of 10% human serum markedly decreased production of TNF-α (to 9% ± 9.5%, *p *< 0.0001) and IL-8 (to 13% ± 25%, *p *< 0.001) (Figure 2). These results are similar to those obtained with PMA-differentiated THP-1 cells.

### Total lipoproteins, HDL, LDL, VLDL, and triglyceride emulsion prevented proinflammatory activity of Mip in primary human differentiated macrophages

**Figure 3:**
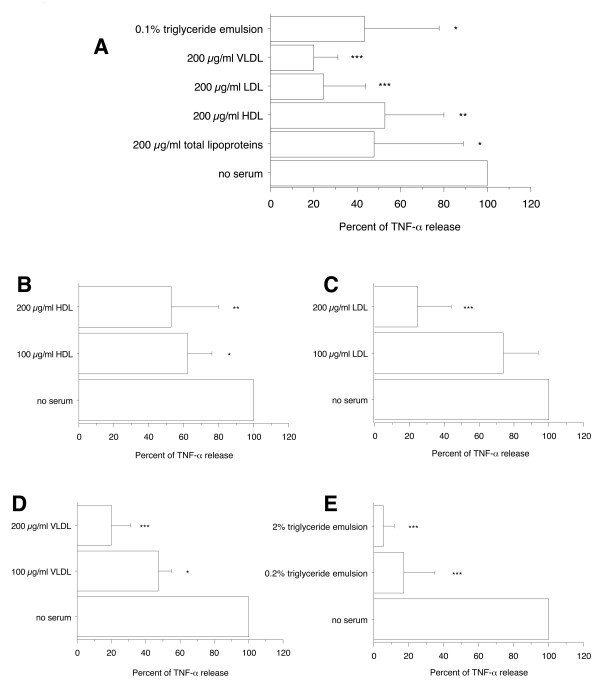
**Effect of total lipoproteins, HDL, LDL, VLDL, and triglyceride emulsion on TNF-α production by primary human differentiated macrophages in response to Mip**. Human monocytes/macrophages (5 × 10^5 ^cells/0.5 ml per well) were differentiated in a 6-day culture in the presence of 2 ng/ml M-CSF. Recombinant Mip at 1 μg/ml was exposed for 30 min to lipoprotein classes or triglyceride emulsion before addition to macrophages. After 4 h stimulation, supernatants were collected and their content in TNF-α were analyzed by ELISA. Each value represents the mean ± SD of triplicates from two experiments. The results are expressed as percent of TNF-α release in the absence of serum. ***: *p *< 0.0001; **: *p *< 0.005, *: *p *< 0.05 determined by comparison with medium alone using Student's t test.

To further examine what serum components can interfere with the proinflammatory activity of Mip, the concentrations of TNF-α were determined in the supernatants of differentiated macrophages after activation with 1 μg/ml of recombinant Mip in the absence of serum and in the presence of 200 μg/ml total lipoproteins, HDL, LDL, or VLDL. In addition, to determine whether circulating triglycerides or the triglyceride component of VLDL might also interfere with Mip-induced TNF-α release, the effect of a 0.1% commercial lipid emulsion composed of long- and medium-chain triglycerides designed for parenteral nutrition (Lipofundin) was examined. The same concentration was tested for the different lipoproteins in order to compare their potential effect. This concentration (200 μg/ml) was either lower than the concentration in normal serum (1400 μg/ml HDL protein, 564 μg/ml LDL protein) or of the same order of magnitude (250 μg/ml VLDL protein) [12,13]. Likewise, the triglyceride concentration (0.1%) was of the same order of magnitude as the concentration in normal serum (300 - 1400 μg/ml). The presence of total lipoproteins, HDL, LDL, VLDL, or 0.1% triglyceride emulsion inhibited significantly the release of TNF-α (to 48 ± 41%, *p *< 0.05, 53 ± 27%, *p *< 0.005, 24 ± 20%, *p *< 0.0001, 20 ± 11%, *p *< 0.0001, and 44 ± 34%, *p *< 0.05, respectively, of the TNF-α concentration present in the absence of serum) (Figure 3A) whereas serum components were devoid of any stimulatory effect on TNF-α production in the absence of Mip (data not shown). The inhibitory effect of HDL, LDL, VLDL, and triglyceride emulsions were dose-dependent (Figures 3B, C, D and 3E). These results confirmed the ability of serum lipids to inhibit Mip-induced TNF-α release and particularly the high potency of LDL and VLDL, explaining probably why FBS which contains no or low concentrations of LDL and VLDL [10,11] is less inhibitory than human serum. In addition they showed that circulating triglycerides or the triglyceride component of VLDL have also the ability to interfer with Mip-induced TNF-α release.

### Human apolipoprotein (apo)A-I, apoB, apoE2, apoE3, and LPS binding protein (LBP) prevented proinflammatory activity of Mip in primary human differentiated macrophages

**Figure 4:**
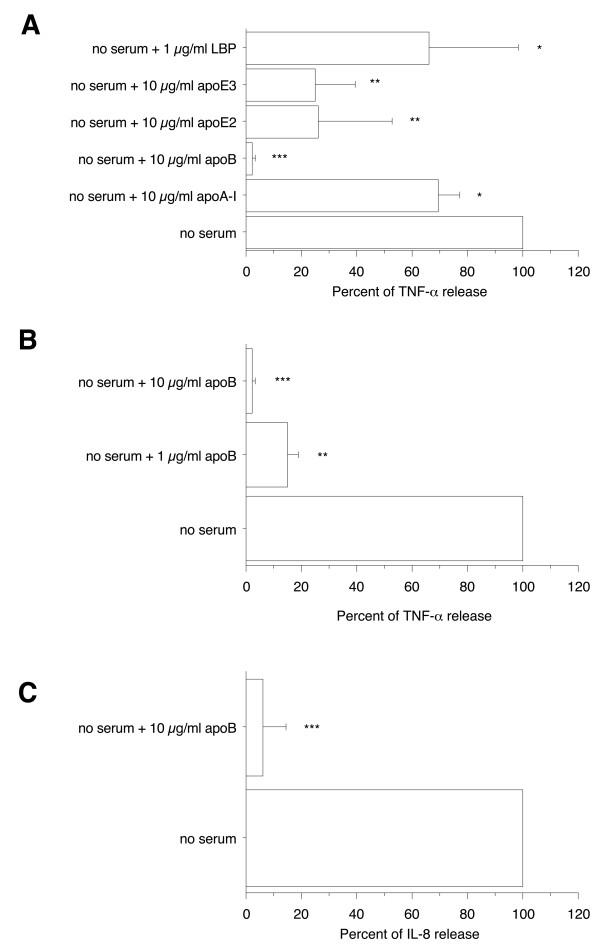
**Effect of human plasma apoA-I, apoB, recombinant apoE2, apoE3, and LBP on TNF-α and IL-8 productions by primary human differentiated macrophages in response to Mip**. Human monocytes/macrophages (5 × 10^5 ^cells/0.5 ml per well) were differentiated in a 6-day culture in the presence of 2 ng/ml M-CSF. Recombinant Mip at 1 μg/ml was exposed for 30 min to apolipoproteins or LBP before addition to macrophages. After 4 h stimulation, supernatants were collected and their content in TNF-α and IL-8 were analyzed by ELISA. Each value represents the mean ± SD of triplicates from two experiments. The results are expressed as percent of TNF-α or IL-8 release in the absence of serum. ***: *p *< 0.0001; **: *p *< 0.005, *: *p *< 0.05 determined by comparison with medium alone using Student's t test.

To further examine the ability of HDL, LDL, and VLDL to inhibit Mip-induced TNF-α production in primary human differentiated macrophages, the effect of purified apoA-I, the major protein component of HDL, and of purified apoB, apoE2, and apoE3, structural components of LDL and VLDL, was examined. To compare their potential effect, the different apolipoproteins were tested at the same concentration (10 μg/ml protein) that was either lower than the concentration in normal serum (1400 μg/ml apoA-I, 810 μg/ml apoB) or of the same order of magnitude (47 μg/ml apoE) [12]. As solubilization of apoB necessitates the presence of deoxycholate, cell cultures were also tested in the presence of the same concentration of this detergent. The presence of 10 μg/ml of apoA-I, apoB, apoE2, or apoE3 significantly inhibited Mip induction of TNF-α release to 70 ± 7%, 2.3 ± 0.8%, 26 ± 27%, and 25 ± 15%, respectively (*p *< 0.05 to < 0.0001), whereas no effect of deoxycholate was observed on Mip-induced TNF-α production. The most potent inhibitor was apoB since practically no TNF-α or IL-8 were detected in the presence of a 10 μg/ml concentration (Figure 4A, 4B, and 4C). The TNF-α inhibition was dose-dependent (Figure 4B). These results agree with the high inhibitory effect observed in the presence of LDL and VLDL (Figure 3A).

The possible inhibition by exogenous human LBP was also examined because LBP is known to circulate in association with HDL, LDL, VLDL, or chylomicrons [14-16]. The concentration tested was 1 μg/ml, which approximately corresponds to the amount of LBP present in 5% normal human serum [17]. The presence of 1 μg/ml LBP slightly inhibited the Mip induction of TNF-α release (66% ± 32%) whereas LBP alone did not affect TNF-α production (Figure 4A).

Overall, these results supported the hypothesis that both protein and lipid serum components interact with Mip lipoprotein and prevent its proinflammatory activity.

### Human serum prevented proinflammatory activity of OspA, *E. coli *LPS, Pam_2_CSK_4, _and Pam_3_CSK_4 _in PMA-differentiated THP-1 cells

**Figure 5:**
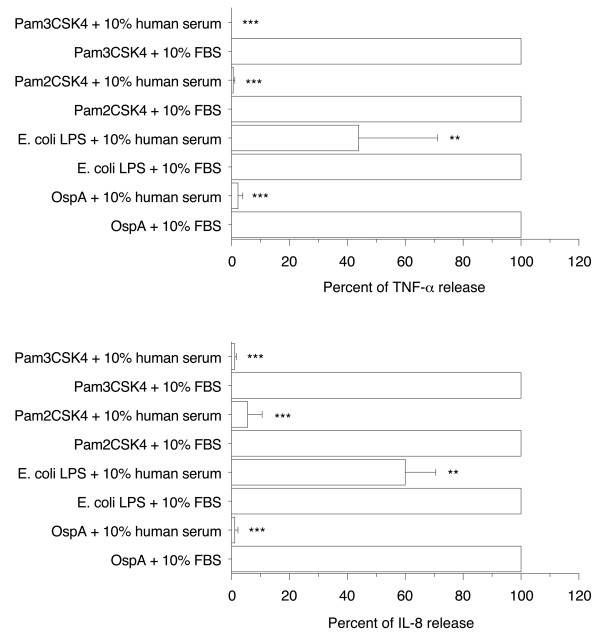
**Effect of 10% FBS and human serum on TNF-α and IL-8 production by PMA-differentiated THP-1 cells in response to OspA, *E. coli *LPS, Pam_2_CSK_4_, or Pam_3_CSK_4_**. Cells (2.5 × 10^5 ^cells/1 ml per well) were cultured with 10 nM PMA for 48 h and then stimulated by recombinant OspA or purified *E. coli *LPS, both at 1 μg/ml, or 0.01 μg/ml Pam_2_CSK_4 _or Pam_3_CSK_4 _in the presence of 10% heat-inactivated FBS or human serum. After 4 h stimulation, supernatants were collected and their content in TNF-α and IL-8 were analyzed by ELISA. Each value represents the mean ± SD of triplicates from four experiments. **: *p *< 0.005, ***: *p *< 0.0001 determined by comparison with 10% FBS using Student's t test.

To investigate whether the inhibitory effect of human serum on Mip-induced TNF-α and IL-8 release was a more general phenomenon, the production of both cytokines was determined in the presence of either 10% FBS or 10% human serum upon stimulation with another bacterial lipoprotein (1 μg/ml OspA), two synthetic lipopeptides (0.01 μg/ml Pam_2_CSK_4 _and Pam_3_CSK_4_), or *E. coli *LPS (1 μg/ml). It was not possible to make a comparison between the absence and presence of human serum because, in contrast to Mip, no or only low production of cytokines was obtained in the complete absence of serum. However, as already mentionned, FBS does not contain VLDL [10,11] and contains lower concentrations of LDL than human serum [10], so the comparison of cytokine production in the presence of FBS and in the presence of human serum allows evaluation of the possible inhibitory effect of LDL and VLDL. Data are reported as percent of the cytokine concentration found in the presence of FBS with 100% corresponding to a median TNF-α concentration of 0.28 ng/ml (IQR 0.23 - 0.73) and to a median IL-8 concentration of 9.8 ng/ml (IQR 5.0 - 14.3) for OspA-stimulated cells, to a median TNF-α concentration of 1.5 ng/ml (IQR 0.9 - 2.0) and to a median IL-8 concentration of 9.9 ng/ml (IQR 4.3 - 15.9) for *E. coli *LPS-stimulated cells, to a median TNF-α concentration of 0.57 ng/ml (IQR 0.13 - 1.19) and to a median IL-8 concentration of 8.4 ng/ml (IQR 6.5 - 11.0) for Pam_2_CSK_4_-stimulated cells, and to a median TNF-α concentration of 1.09 ng/ml (IQR 0.71 - 1.14) and to a median IL-8 concentration of 18 ng/ml (IQR 14 - 21) for Pam_3_CSK_4_-stimulated cells. Compared to cytokine release observed in the presence of 10% FBS, the production of TNF-α and IL-8 induced by OspA, Pam_2_CSK_4_, or Pam_3_CSK_4 _were completely inhibited in the presence of 10% human serum but not the production induced by *E. coli *LPS which were only significantly inhibited (to 44 ± 27% for TNF-α and 60 ± 10% for IL-8, *p *< 0.005 in both cases)(Figure 5).

### Human serum prevented proinflammatory activity of OspA, *E. coli *LPS, Pam_2_CSK_4_, and Pam_3_CSK_4 _in primary human differentiated macrophages

**Figure 6:**
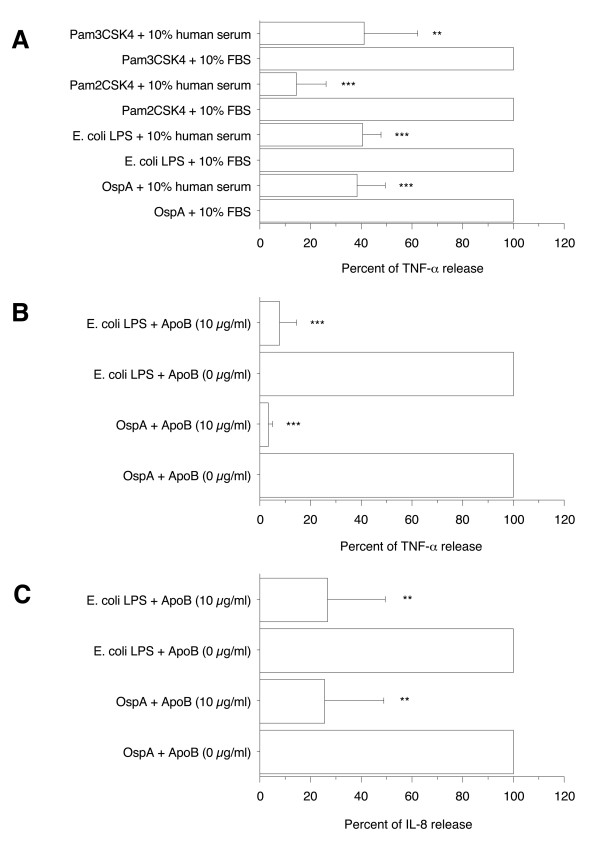
**Effect of 10% FBS and human serum on TNF-α productions by primary human differentiated macrophages in response to OspA, *E. coli *LPS, Pam_2_CSK_4_, and Pam_3_CSK_4 _and effect of 10 μg/ml apoB on TNF-α and IL-8 production induced by OspA and *E. coli *LPS**. Human monocytes/macrophages (5 × 10^5 ^cells/0.5 ml per well) were differentiated in a 6-day culture in the presence of 2 ng/ml M-CSF and then stimulated by recombinant OspA or purified *E. coli *LPS, both at 1 μg/ml, or 0.01 μg/ml Pam_2_CSK_4 _or Pam_3_CSK_4 _in the presence of 10% heat-inactivated FBS or human serum (A) or in the presence of 10 μg/ml human plasma apoB (B and C). After 4 h stimulation, supernatants were collected and their content in TNF-α and IL-8 were analyzed by ELISA. The results are expressed as percent of TNF-α or IL-8 release in presence of 10% FBS. Each value represents the mean ± SD of triplicates from two experiments. **: *p *< 0.005, ***: *p *< 0.0001 determined by comparison with 10% FBS using Student's t test.

To extend our observations, the effect of 10% human serum was also compared to 10% FBS in primary human differentiated macrophages. In contrast to PMA-differentiated THP-1 cells, the production of TNF-α induced by OspA, Pam_2_CSK_4_, or Pam_3_CSK_4 _was not completely inhibited in the presence of 10% human serum. However significant inhibitions were still observed (to 38 ± 11% for OspA, 15 ± 12% for Pam_2_CSK_4_, and 41 ± 21% for Pam_3_CSK_4_, *p *< 0.0001 to < 0.005) as well as for *E*. coli LPS-stimulated cells (40 ± 8%, *p *< 0.0001)(Figure 6A). Overall, these results suggest a role for LDL and VLDL in the inhibition of cytokine production observed in the presence of human serum.

### Human apoB prevented proinflammatory activity of OspA and *E. coli *LPS

To further examine the possible role of LDL and VLDL in inhibition of proinflammatory activity of these bacterial compounds, the ability of purified apoB, the main structural component of LDL and VLDL, to alter the proinflammatory activity of OspA and *E. coli *LPS was next investigated. At a concentration of 10 μg/ml, apoB was found to markedly inhibit the TNF-α and IL-8 production induced by 1 μg/ml OspA or *E. coli *LPS (to 3 ± 2% and 8 ± 6%, respectively for TNF-α production, *p *< 0.0001 in both cases and to 26 ± 23% for IL-8 production, *p *< 0.005, in both cases)(Figure 6B and 6C). This inhibitory effect of apoB was concentration-dependent (data not shown). These data demonstrated a general ability of purified apoB to inhibit proinflammatory activity of bacterial components.

### Low sequence homologies were found between apoA-I, apoE, and C- terminal sequence of apoB

**Figure 7:**
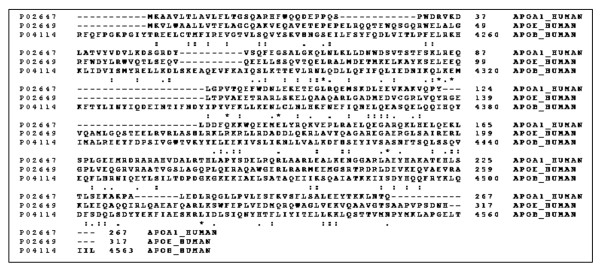
**Sequence alignment of human apoA-I (accession number P02647, 267 residues), apo B (P04114, 4563 residues), and apoE (P02649, 317 residues) as performed by the CLUSTAL multiple alignment program**. Asterisks indicate identities and colons (conserved substitutions) and periods (semiconserved substitutions) indicate similarities in the lines below the sequences.

Because apoA-I, apoE, and apoB were shown to inhibit TNF-α release in Mip-stimulated cells (Figure 4A), homologies among the three apolipoprotein sequences were studied to identify possible residues in the apolipoproteins that account for the neutralization of Mip. The CLUSTALW multiple sequence alignment program was used and similarities were found between apoA-I, apoE, and C- terminal part of apoB. However, sequence homologies were low since only 7 (2%) invariant residues, 54 (15%) conserved substitutions, and 28 (8%) semiconserved substitutions were found in 362 residues overlap (Figure 7). With pairwise alignments, the best score (114) was obtained between apoA-I and apoE sequences which resulted in 20.7% identity in 164 residues overlap. When pairwise alignments were performed with apoB, lower scores were obtained: 51 with apoE (20.9% identity in 43 residues overlap) and 46 with apoA-I (20.8% identity in 72 residues overlap). These results suggest that the interactions between apolipoproteins and Mip do not involve particular residues but might rather involve specific domains as reported by Segrest et al. [18]. Thus, the hydrophobic surfaces of apolipoproteins might interact with acyl chains of the lipid part of Mip, rendering it biologically inactive and significantly less stimulatory to macrophages because the lipid part of Mip is responsible of its proinflammatory activity [2].

### Serum lipids inhibited the production of IL-8 induced by Mip and OspA in HEK-293 cells expressing human TLR1/2

**Figure 8:**
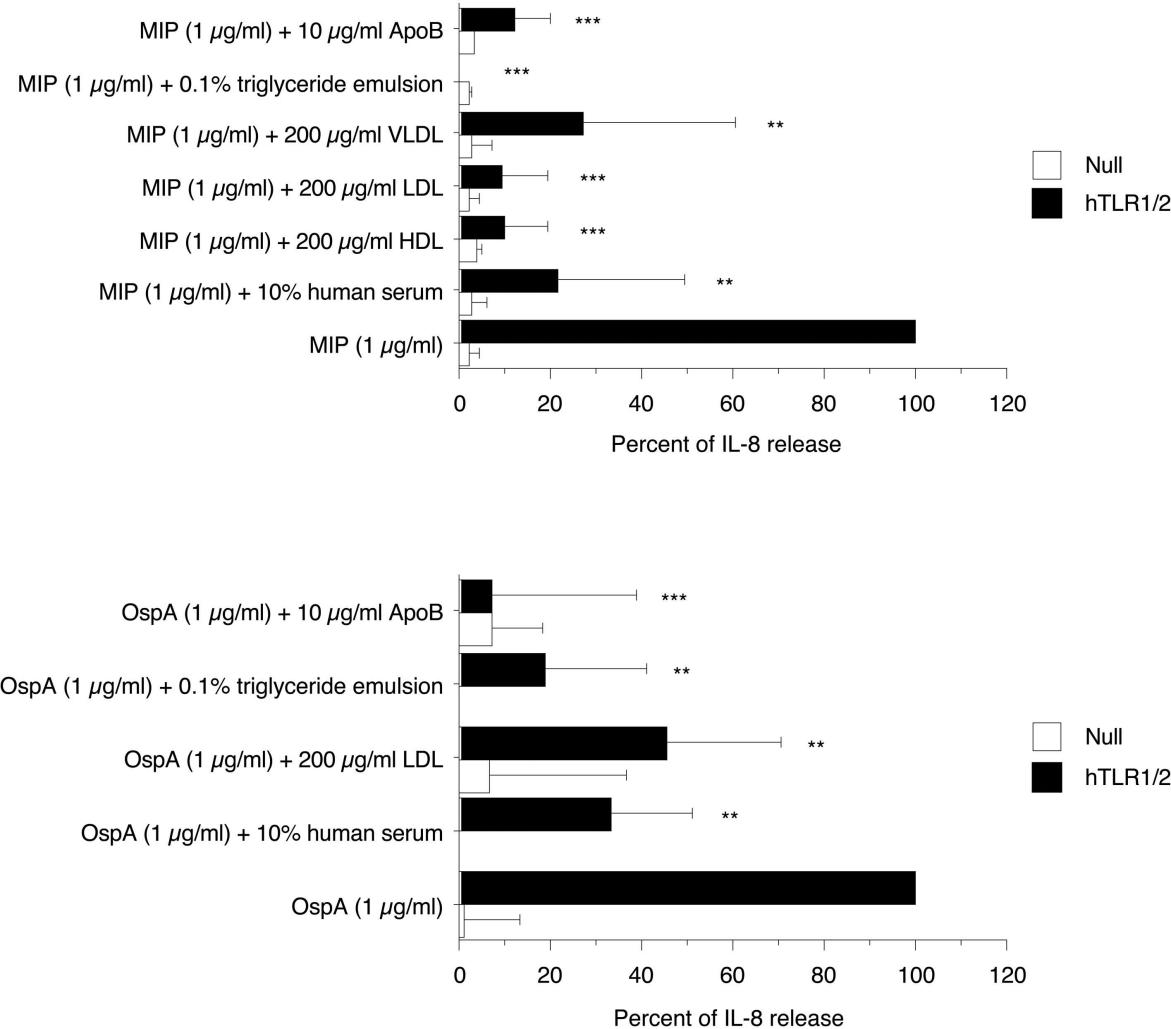
**Effect of human serum, LDL, triglyceride emulsion, and apoB on the production of IL-8 by HEK-293 cell line expressing human TLR1/2, upon Mip and OspA activation**. Null and hTLR1/2 cells were stimulated with 1 μg/ml Mip or OspA for 24 h in the presence or absence of serum lipids. Culture supernatants were collected and IL-8 content was analyzed. Background values (unstimulated) were subtracted. Each value represents the mean ± SD of triplicates from two experiments. **: *p *< 0.005, ***: *p *< 0.0001 determined by comparison with bacterial component alone using Student's t test.

To gain further insight into the mechanism involved in serum factor-driven attenuation of bacterial lipoprotein proinflammatory activity and more precisely examine the ability of serum lipids and lipoproteins to render bacterial lipoproteins invisible to signaling receptors, the effect of 10% human serum, 200 μg/ml HDL, LDL, or VLDL, 0.1% triglyceride emulsion, and 10 μg/ml apoB was tested in HEK-293 cell lines expressing human TLR1/2, recently identified as the main receptors involved in the recognition of Mip [2]. HEK-293 cells stably transfected with either the empty plasmid (293-Null) or human TLR1/2 genes were stimulated with 1 μg/ml Mip or OspA. The IL-8 production obtained in Mip-stimulated HEK-293 cells expressing human TLR1/2 was markedly inhibited by the presence of serum lipids (to 22 ± 28%, 10 ± 10%, 10 ± 10%, 27 ± 34%, 0%, and 12 ± 8% (*p *< 0.005 and < 0.0001) in the presence of 10% human serum, 200 μg/ml HDL, LDL, or VLDL, 0.1% triglyceride emulsion, or 10 μg/ml apoB, respectively. Similar results were obtained in OspA-stimulated HEK-293 cells expressing human TLR1/2 (Figure 8) suggesting that these factors interfered with the binding of Mip and OspA to TLR1/2.

## Discussion

Different bacteria or bacterial components are known to interact with serum lipoproteins or apolipoproteins. The best characterized interactions are those between *E. coli *LPS and HDL [19,20] but also LDL, VLDL, and chylomicrons [21,22] as well as purified human apoA-I [22-24], apoB [23], and apoE [25,26]. Lipoteichoic acid has also been shown to interact with several plasma lipoproteins [9,27] as well as *Staphylococcus aureus *α-toxin [28], *Porphyromonas gingivalis *[29], *Vibrio vulnificus *cytolysin [30], and *Streptococcus pyogenes *collagen-like protein Scl1 [31]. Very few studies have described interactions between bacterial lipopeptides or lipoprotein and serum components with one report about the binding of lipopeptides from *Mycoplasma arthritidis *to apoA-I [32] and another one about the interaction of pH6-Ag, a lipoprotein of *Yersinia pestis*, with apoB [33]. The present study adds new lipoproteins to the list of bacterial components known to bind serum lipoproteins or apolipoproteins. Indeed, human serum was found to markedly inhibit Mip proinflammatory activity. When tested independently, total lipoproteins, HDL, LDL, VLDL or a triglyceride emulsion all had an inhibitory effect, LDL and VLDL being the most potent. When several apolipoproteins were tested without their physiological lipid complement such as purified apoA-I, apoB, apoE2, and apoE3, all showed an inhibitory effect, apoB being the most potent. This common inhibitory effect cannot be attributed to sequence similarity between the different apolipoproteins but rather to similarity in their lipid-associating domains. Indeed, apoA-I and E are characterized by an abundance of amphipathic α-helices [34] responsible for their lipid binding character [35] and it has been shown that the α2 and α3 domains in apoB-100, corresponding to the two major apoB-100 lipid-associating domains, are homologous to certain amphipatic helix-containing regions of apoA-I and apoE [18]. All these amphipatic domains might interact with the acyl chains of the lipid-modified cysteine at the amino-terminus of Mip.

The possible involvement of LBP in Mip inactivation was also investigated but was found to be poor compared to apoB ability. This result agrees with the absence of LBP effect on cytokine production induced by *B. burgdorferi *OspA, another lipoprotein [36,37]. In addition, human serum was able to decrease TNF-α production induced by synthetic triacylated and diacylated lipopeptides, OspA, and *E. coli *LPS and apoB was able to inactivate OspA and *E. coli *LPS. Results obtained with HEK-293 cell lines expressing human TLR1/2 showed that serum factors attenuated proinflammatory activity of both Mip and OspA through their binding to TLR1/2. These results allow us to hypothesize that an attenuation of bacterial lipoprotein proinflammatory activity may occur in blood and in all body compartments where serum lipoproteins are present. This observation may have an important impact because bacterial lipoproteins are produced by the complete spectrum of bacterial pathogens [38] and have been implicated in inflammatory processes and in pathogenesis of several important bacterial infections, including *Leptospira interrogans *[39], *Mycobacterium tuberculosis *[40], *Treponema pallidum *[41], *Listeria monocytogenes *[42], and *Borrelia burgdorferi *[41]. Lipoproteins can be spontaneously released from membranes when cells are lysed [43,44] and treatment of bacteria with antibiotics has been shown to significantly enhance bacterial lipoprotein release [45]. Thus, bacterium-associated lipoproteins have been found in culture supernatants [45,46], infected tissues [47], or the bloodstream in gram-negative sepsis [47]. For whole bacteria, the serum lipoprotein deposition on the bacterial surface could prevent recognition of the pathogen by the host defense and be detrimental by impairing the antibacterial response but this mechanism might also be of benefit by preventing ongoing excessive inflammation. However, if this mechanism could play a role in whole blood, in extravascular compartments or at mucosal surfaces, where serum lipoprotein concentrations are lower, bacterial lipoproteins can have proinflammatory effects similar to those described in vitro in the absence of serum. For Chlamydiae that are not primarily bloodstream infectious agents but rather bacteria infecting lung, urogenital system or eyes, i.e. serum-free compartments, the recognition and responses would be much more sensitive than in the circulation. However, plasma components can leak into the sites of infection and antagonize the stimulatory effect induced by bacteria. For example, cervico-vaginal fluid [48,49] and tears [50] have been shown to contain apoA-I. As previously described for LPS [51], two pathways would coexist: one leading to host cell activation and involving bacterial lipoprotein/lipopeptide interaction with CD14 and TLR2/1/6, and another leading to deactivation and involving bacterial lipoprotein/lipopeptide sequestration by serum lipoproteins. Depending on body compartments, bacterial lipoproteins should be viewed as a double-edged sword in host-pathogen interactions: they can serve both as signal recognized by the host to activate its defenses and limit infection and as agents causing excessive host damage by the pathogen in some situations.

Concerning the mechanism involved in bacterial lipoprotein inactivation by apolipoproteins, it is possible to hypothesize that this is effected by the constitution of micelles between the hydrophobic structure of apolipoproteins and the hydrophobic part of bacterial lipoproteins. For the inactivation by triglycerides, it is probably mediated by a lipid-lipid interaction with the fatty acyl chains of the lipid portion of bacterial lipoproteins. The lipid component of bacterial lipoprotein, known to be the moiety causing monocyte/macrophage activation [2], would be sheltered or sequestered and unable to bind to cell receptors. It has been shown that apoB-containing lipoproteins prevented binding of pH6-Ag to THP-I monocyte-derived macrophages [33]. The present study has shown that serum lipoproteins inhibited TLR1/2-mediated proinflammatory response to two bacterial lipoproteins, Mip and OspA.

## Conclusions

In conclusion, this study shows that a close relationship exists in vitro between serum and bacterial lipoproteins that is able to influence proinflammatory activity of bacterial components.

## Methods

### Reagents

Recombinant Mip lipoprotein was purified as previously described [52] and subsequently treated by polymyxin B-agarose (Sigma-Aldrich, Buchs, Switzerland) [2]. Recombinant *Borrelia burgdorferi *outer surface protein A (OspA) was purchased from Biodesign International (Milan Analytica, La Roche, Switzerland). Racemic Pam_3_CSK_4 _(Pam_3_-Cys-Ser-Lys_4_-OH) and Pam_2_CSK_4 _(Pam_2_-Cys-Ser-Lys_4_-OH) used as synthetic triacylated and diacylated control lipopeptides, respectively, were obtained from EMC Microcollections (Tuebingen, Germany). LPS from *Escherichia coli *serotype O55:B5 was purchased from Sigma-Aldrich and repurified [2]. Human serum and FBS was from Invitrogen (Basel, Switzerland). Delipidated FBS was purchased from Sigma-Aldrich. Long- and medium-chain triglyceride emulsion (Lipofundin; 20%, w/v) was from B. Braun (Melsungen, Germany). Human plasma apoA-I and B were from Calbiochem (Merck Biosciences, VWR International, Dietikon, Switzerland). Recombinant apoE2 and E3 were from Invitrogen. Recombinant LBP was obtained from Biometec (Greifswald, Germany).

### Preparation of lipoproteins

Total serum lipoproteins were isolated from human serum by salt gradient ultracentrifugation at d < 1.21 gm/cm^2^. HDL, LDL, and VLDL were isolated by sequential density ultracentrifugation as described previously [53,54] and dialyzed for 48 h against PBS or RPMI. Protein concentrations were determined by using a micro-bicinchoninic acid protein assay kit (Pierce, Perbio Science, Switzerland) using BSA as a standard.

### THP-1 cell culture

The method of THP-1 cell culture was previously described [2]. Briefly, THP-1 cells were grown in RPMI 1640 medium. For monocytic differentiation, they were seeded in 24-well flat-bottom tissue culture plates at a density of 2.5 × 10^5 ^cells/1 ml per well and allowed to adhere and differentiate 48 h at 37°C in the presence of 10 nM PMA (Sigma-Aldrich). After repeated washing with RPMI 1640, PMA-differentiated THP-1 cells were stimulated at 37°C with indicated stimuli. Cell-free supernatants were harvested after 4 h (or indicated time periods) of incubation and kept at -70°C until cytokine measurements.

### Human differentiated macrophage culture

The study protocol was approved by Institutional Ethics Committee (Geneva University Hospital, Switzerland). Informed consent was obtained from all subjects. The method of monocyte/macrophage preparation was previously described [2]. Briefly, peripheral blood mononuclear cells from healthy blood donors were isolated by density gradient centrifugation with Ficoll-Hypaque (Amersham Biosciences, GE Healthcare Europe GmbH, Otelfingen, Switzerland). Monocytes/macrophages were separated by aggregation, gradient of FBS, and rosetting. Macrophages were generated by differentiation of purified monocytes/macrophages using a 6-day culture in the presence of 2 ng/ml macrophage-colony-stimulating factor (M-CSF) (R&D Systems, Abingdon, UK). Monocytes/macrophages were seeded into 24-well flat-bottom tissue culture plates at a concentration of 5 × 10^5 ^cells/0.5 ml per well in RPMI 1640 containing 2 mM GlutaMAX I, supplemented with 100 U/ml penicillin, 0.1 mg/ml streptomycin, and heat inactivated (30 min at 56°C) endotoxin-free 10% (v/v) human serum (Invitrogen). The medium was refreshed regularly. After 6-day differentiation, cultures were washed five times with medium without serum. Recombinant Mip was exposed for 30 min to human serum, isolated lipoproteins, or triglyceride emulsion before addition to macrophages. After 4 h stimulation at 37°C with indicated stimuli, cultures were centrifuged at 400 × *g *for 10 min at 4°C and cell-free supernatants were collected and stored at -70°C until TNF-α measurements.

### Cytokine measurements

Extracellular release of TNF-α and IL-8 was determined by a sandwich ELISA technique using the DuoSet ELISA Development Systems (R&D), according to the manufacturer's instructions. The ELISA detection limits were 2 pg/ml.

### Sequence alignments

The sequences of apoA-I, apoB, and apoE were found in the Swiss-Prot/TrEMBL database. To study homologies among the three apo sequences, the CLUSTALW multiple sequence alignment program was used. To study homologies between two sequences, the SIM binary sequence alignment program [55] (available at http://www.expasy.org/tools/sim-prot.html) was used with Blosum62 as a comparison matrix.

### Response of TLR1/2 cell lines

Nonphagocytic HEK-293 cells stably transfected with either the empty plasmid (293-Null) or human TLR1/2 genes were purchased from InvivoGen (LabForce, Nunningen, Switzerland) and maintained in Dulbecco's Modified Eagle Medium (Invitrogen) supplemented with 4.5 g/l glucose, 10% FBS, 100 U/ml penicillin, 0.1 mg/ml streptomycin, and 10 μg/ml blasticidin S (InvivoGen). For stimulation experiments, stable transfected cells were seeded into individual wells of a 48-well flat-bottom tissue culture plate at a concentration of 3 × 10^5 ^cells/0.3 ml per well of complete medium and allowed to adhere overnight. The following day, fresh medium was added and the cells were stimulated with indicated stimuli for 24 h. Culture supernatants were collected and IL-8 content was analyzed.

### Statistical analysis

Statistical analyses were performed using the Student's *t *test with the SPSS statistical software (for Macintosh, v.10). Differences were considered significant at *p *< 0.05.

## Authors' contributions

SB: conception, design, analysis and interpretation of data, drafting of the manuscript and final approval of the manuscript. RWJ: preparation of lipoproteins, final approval of the manuscript. CG has made substantial contributions in revising the manuscript critically. All authors read and approved the final manuscript.
